# Expression and clinical significance of Caveolin-1 in prostate Cancer after transurethral surgery

**DOI:** 10.1186/s12894-018-0418-4

**Published:** 2018-11-13

**Authors:** Xiaoming Wang, Zhigui Liu, Zhanbin Yang

**Affiliations:** 1 Department of Urology, The First Affiliated Hospital of Guangxi Medical University, Nanning, 530021 China; 2 Department of Urology, The Second Affiliated Hospital of Guangxi Medical University, No 166 DaXueDong Road, Nanning, 530007 Guangxi China

**Keywords:** Prostate cancer, Benign prostatic hyperplasia, Caveolin-1, Immunohistochemistry

## Abstract

**Background:**

Prostate cancer is a common malignancy of the male genitourinary system that occurs worldwide. The current research aims to investigate caveolin-1 expression in prostate cancer tissue and its relationship with pathological grade, clinical pathologic staging, and preoperative prostate-specific antigen (PSA) levels.

**Methods:**

From January 2012 to December 2014, samples from 47 patients with prostate cancer who had received transurethral prostatic resection (TURP) and 20 patients with benign prostatic hyperplasia were collected at the First Affiliated Hospital of Guangxi Medical University. Caveolin-1 was detected by streptavidin-perosidase (SP) immunohistochemical staining in pathological tissue slices. The results were statistically analyzed for pathological grade, clinical stage, and preoperative PSA level.

**Results:**

The expression of caveolin-1 was significantly higher in prostate cancer samples than in benign prostatic hyperplasia samples (*P* < 0.05), and caveolin-1 expression was significantly different among the pathological grades of poorly, moderately and well-differentiated prostate cancer (*P* < 0.05). The difference in caveolin-1 expression was significant for different clinical stages (T1-T2 and T3-T4) of prostate cancer (P < 0.05). The difference in caveolin-1 expression was not significant among samples with different preoperative PSA levels (0–10, 10–100 and > 100 μg/L) (*P* > 0.05).

**Conclusions:**

Caveolin-1 is closely related to the pathological grade and clinical stage of prostate cancer after transurethral surgery, and it may be a novel tumor marker for prostate cancer. The expression of caveolin-1 is not associated with preoperative serum PSA levels.

## Background

Prostate cancer is a common malignancy of the male genitourinary system. In recent years, there has been a continuous rise in the incidence of prostate cancer cases, and it is becoming the first cancer to threaten male health [1, 2]. Screening for prostate cancer relies mainly on PSA testing. However, because of low PSA specificity and the very low serum PSA levels in some patients with prostate cancer, there is a possibility of missed diagnosis [3, 4]. Therefore, finding new screening markers is essential for providing powerful evidence for the early detection, early diagnosis, and early treatment of prostate cancer [5].

At present, the pathogenesis and relevant molecular biological basis of prostate cancer are not yet fully understood [6]. Recent studies have proved that caveolin-1 is associated with the incidence and progression of prostate cancer [7], but the specific mechanism is not clear.

Caveolin (Cav) is a special vesicle-like structure on the surface of the cell membrane and is an important structural-functional protein. It has been found that mammals have three caveolin genes (caveolin-1, caveolin-2 and caveolin-3). Caveolin-1 and caveolin-2 are widely present in a variety of normal human cells, and caveolin-3 is expressed specifically in muscle. Caveolin interacts with specific lipids, such as glycosphingolipids or cholesterols, thereby leading to the formation of caveolae. In the sub-cell, caveolin-1 is positioned at the cytoplasm, mitochondria, caveolae, and other related secretion channels [8]. Caveolin-1 is involved mainly in the interaction of signaling molecules and the regulation of signaling channels, as well as the regulation of cell proliferation, differentiation, migration, apoptosis, and angiogenesis signaling pathways. Caveolin-1 is also closely related to the occurrence, progression, invasion, and metastasis of tumors [9].

A large number of studies have shown that caveolin-1 is expressed at a low level in most tumors and is present as a tumor suppressor gene [10]. However, it has been found that caveolin-1 protein expression is not consistent in different tumor tissues and cell lines; in a small number of malignant tumors, specifically urinary tract tumors (prostate cancer, kidney cancer, and bladder cancer), it is highly expressed and is thus a gene related to cancer and cancer metastasis [11].

In this study, caveolin-1 was detected in pathological tissue slices of prostate cancer by streptavidin-peroxidase (SP) immunohistochemical staining. Statistical analysis was performed on the immunohistochemistry results for the tissue slices to investigate the correlation of caveolin-1 with the prostate cancer pathological grade, clinical stage, and preoperative serum PSA level to provide new information for the clinical diagnosis and treatment of prostate cancer.

## Materials and methods

### General information

Inpatients who received transurethral resection of prostate in the Urology Department of the First Affiliated Hospital of Guangxi Medical University between January 2012 and December 2014 were included in this study. The inclusion criteria were patients who had undergone surgical resection for prostate cancer and had benign prostatic hyperplasia that had been diagnosed pathologically. The exclusion criteria included 1) patients with non-prostate cancer and benign prostatic hyperplasia; 2) patients without pathological reports; 3) patients with incomplete clinical data; and 4) patients who had received preoperative radiotherapy, chemotherapy or endocrine treatment. Forty-seven samples from patients with transurethral electrocision of the prostate and a pathological diagnosis of prostate cancer were included in the experimental group. In the experimental group, the patients’ ages ranged from 55 to 91, and the average age was 71.15 ± 9.60 years old. In addition, 20 patients with benign prostatic hyperplasia were selected as the control group. In the control group, the patients’ ages ranged from 48 to 79, and the average age was 69.63 ± 8.24 years old. All specimens were from patients who were admitted to the Urology Department of the First Hospital Affiliated of Guangxi Medical University after transurethral electrocision of the prostate. The clinical data collected from all patients included general information (including name, age, inpatient number, admission diagnosis, and contact information) and the results of auxiliary examination, including the preoperative serum PSA level, tumor node metastasis (TNM) staging, pathological findings (including Gleason score), and treatment.

### Immunohistochemical detection

Cav-1 protein was detected by the standard streptavidin-peroxidase (SP) immunohistochemical staining method. The tissue slices were processed and incubated with a rabbit anti-human caveolin-1 polyclonal antibody (1:200 dilution; cat. no. SC-894; Santa Cruz Biotechnology Inc., CA, USA) overnight at 4 °C; then, the slices were incubated with biotin-labeled goat anti-rabbit IgG (1:200; Histostain-SP Kit; cat. no. SPN-9001; Zsbio, Beijing, China) at room temperature for 20 min; next, the slices were incubated with peroxidase-labeled streptavidin at room temperature for 20 min and stained with a Tris-HCl solution containing 0.02% 3,9-diaminobenzidine for 5 to 7 min; finally, the slices were stained with hematoxylin, washed with water, dehydrated, removed, sealed, detected and photographed under a microscope.

### Result analysis

Positive results were analyzed using an Olympus optical microscope, and double-blind randomized observations were made by two chief physicians. Positive caveolin-1 expression was located in the cell membrane and cytoplasm and was indicated by yellowish-brown or brown granules. The scores were calculated according to the staining intensity of positive-staining cells and the number of positive-staining cells (percentage of positive cells) in the tissue slice. (1) The staining intensity was calculated as follows: negative - 0 points; weak positive - 1 point; positive - 2 points; and strong positive - 3 points. For the tissue slices, the staining intensity was calculated as follows: colorless - 0 points; light yellow - 1 point; yellowish-brown - 2 points; and brown - 3 points. (2) The percentage of positive cells were calculated as follows: < 5% - 0 points; ≥ 5 to 25% - 1 point; > 25 to 50% - 2 points; > 50 to 75% - 3 points; and > 75% - 4 points. The final score was calculated by multiplying the staining intensity score by the positive cell percentage. When the final score was ≥4 points, it was considered positive; when the final score was < 4 points, it was considered negative.

### Statistical analysis

All statistical analyses were performed using the SPSS 16.0 software (SPSS Inc., Chicago, IL). Group comparisons were performed using the Chi-square test. Differences were considered statistically significant when *p* < 0.05.

## Results

### Experimental group: Clinical and pathological data

As shown in Table 1, in the experimental group, 19 patients (40.4%) were younger than 70 years old, and 28 patients (59.6%) were older than or equal to 70 years old. The preoperative PSA level was 0–10 ng/ml in 6 cases (12.8%), 10–100 ng/ml in 22 cases (46.8%), and > 100 ng/ml in 19 cases (40.4%). The clinical stage was T1-T2 in 26 cases (55.3%) and T3-T4 in 21 cases (44.7%). According to the 2016 World Health Organization (WHO) classification of tumors of the urinary system and male genital organs and the risk classification, which is based on the D’amico classification system, the Gleason score was categorized as follows: low risk 2–6, intermediate risk 7, and high risk 8–10. This study found 2–6 points in 11 cases (23.4%), 7 points in 16 cases (34.0%), and 8–10 points in 20 cases (42.6%).

**Table 1:** Clinical and pathological data in 47 cases of patients with prostate cancer

	Item	Cases (percentage)
Age	< 70	19 (40.4%)
	≥70	28 (59.6%)
Preoperative PSA	0–10	6 (12.8%)
level (ng/ml)	10–100	22 (46.8%)
	> 100	19 (40.4%)
Clinical stage	T1-T2	26 (55.3%)
	T3-T4	21 (44.7%)
Gleason score	2–6	11 (20%)
	7	16(37.4%)
	8–10	20(42.6%)

### Tissue expression of caveolin-1 in prostate cancer and benign prostatic hyperplasia

**Fig. 1:**
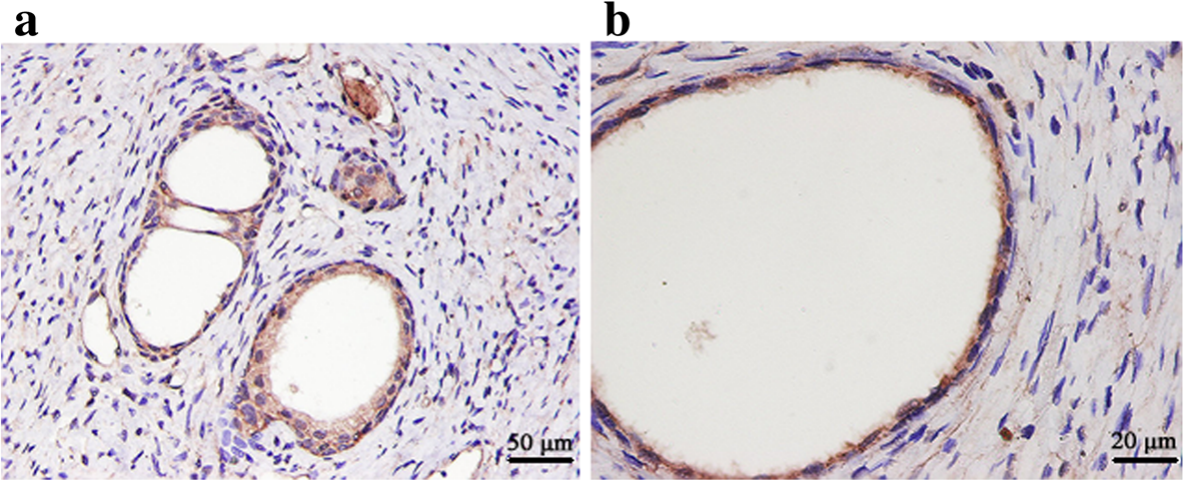
Caveolin-1 expression in benign prostatic hyperplasia. a: SP × 200; b: SP × 400

Caveolin-1 positive staining was found in the cell membrane and cytoplasm of prostate cancer (PCa) cells, presenting as yellowish-brown or brown particles. In 47 cases of prostate cancer, positive expression was found in 29 cases, and negative expression was found in 18 cases; the positive expression rate was 61.70%. In 20 cases of benign prostatic hyperplasia, positive expression was found in 2 cases, and negative expression was found in 18 cases; the positive expression rate was 10.0% (Fig 1). The difference between the two groups was statistically significant (Table 2).

**Table 2:** Tissue caveolin-1 expression in prostate cancer and benign prostatic hyperplasia

	Cases	Positive	Negative	Positive rate	X^2^	*P* value
Prostate cancer	47	29	18	61.70%	15.085	0.000
Benign prostatic hyperplasia	20	2	18	10%		

### Correlation of the expression of caveolin-1 and pathological grading

**Fig. 2:**
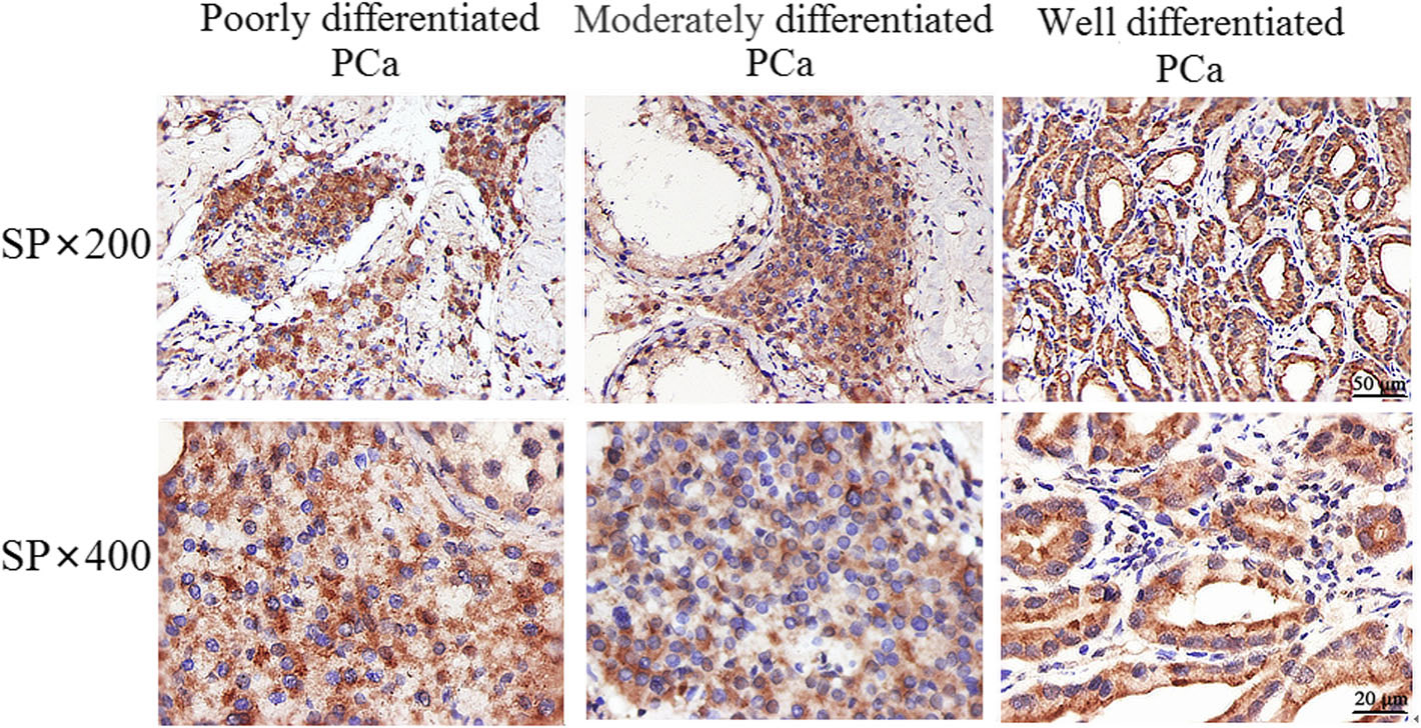
Caveolin-1 expression in prostate cancer tissues with different pathological grades

As shown in Fig. 2, the positive expression rate of caveolin-1 in PCa tissue was 61.70%. The positive expression rate of caveolin-1 was 80.0% in poorly differentiated PCa tissue, 68.8% in moderately differentiated PCa tissue, and 18.2% in well-differentiated PCa tissue. The higher the differentiation of the prostate cancer tissue was, the stronger the staining intensity of caveolin-1, the higher the percentage of positive cells, and the higher the positive rate were. The inter-group difference was statistically significant (Table 3). Therefore, prostate cancer is closely related to caveolin-1 since prostate cancer of a high pathological grade was often accompanied by the high expression of caveolin-1.

**Table 3:** Correlation of caveolin-1 expression with pathological grade of prostate cancer

Pathological grade	Cases	Positive	Negative	Positive rate	X^2^	*P* value
poorly differentiation	20	16	4	80.0%	8.773	0.012
Moderately differentiation	16	11	7	68.8%		
Well differentiation	11	2	7	18.2%		

### Correlation of the expression of caveolin-1 and the clinical stage of prostate cancer

The positive rates of caveolin-1 expression in T1-T2 and T3-T4 PCa tissues were 46.15 and 80.95%, respectively. The expression rate of caveolin-1 increased with the clinical stage. The difference between the two groups was statistically significant (Table 4), suggesting that caveolin-1 was related to prostate cancer invasion (*P* < 0.05).

**Table 4:** Correlation of caveolin-1 expression with clinical stage of prostate cancer

Clinical grade	Cases	Positive	Negative	Positive rate	X^2^	P value
T1-T2	26	12	14	46.15%	5.953	0.015
T3-T4	21	17	4	80.95%		

### Correlation of the expression of caveolin-1 and the preoperative PSA level in patients with prostate cancer

The positive expression rates of caveolin-1 in preoperative PSA groups (0–10 μg/L, 10–100 μg/L, and > 100 μg/L) were 16.67, 68.18 and 68.42%, respectively. The difference was not statistically significant (*P* > 0.05) (Table 5), suggesting that caveolin-1 and preoperative PSA levels in patients may be two independent markers.

**Table 5:** Correlation of caveolin-1 expression with preoperative PSA levels in patients with prostate cancer

Preoperative PSA level	Cases	Positive	Negative	Positive rate	X^2^	*P* value
0–10	6	1	5	16.67%	5.904	0.052
10–100	22	15	7	68.18%		
>100	19	13	6	68.42%		

## Discussion

The occurrence and progression of prostate cancer as well as its surrounding infiltration plus distant metastasis are closely related to abnormal gene and protein expression. In cases where the tumor suppressor gene is an inactivator and the oncogene is an activator, protein expression will be abnormal, resulting in the deregulation of cell differentiation and growth disorders and eventually leading to the occurrence of prostate cancer [12].

The expression of caveolin-1 is low in most tumors, indicating its role as a tumor suppressor, but the specific mechanism is still unclear. It is generally believed that caveolin-1 can inhibit the malignancy of cells by inhibiting the activity of the cyclin D1 gene promoter, thereby inhibiting the MAPK pathway and the phosphorylation of the Src tyrosine kinase [13]. The reduced expression of caveolin-1 can promote the activation of gastric cancer-related fibroblasts, resulting in gastric cancer [14]. The expression of caveolin-1 in leukemia HL-60 cells can inhibit tumor cell proliferation, induce apoptosis, block PI3K/AKT signaling pathway activation and enhance chemotherapeutic agent sensitivity [15]. The expression of caveolin-1 is also reduced in many primary tumors, such as colorectal cancer, ovarian cancer, and breast cancer [10, 16, 17]. The above evidence suggests that the reduced expression of caveolin-1 is closely related to tumorigenesis. In contrast to the above studies, the expression of caveolin-1 was significantly higher in some tumor tissues than in normal tissues. Tse et al. [18] found that caveolin-1 was not expressed in normal livers and normal liver cell lines, but it is positively expressed in hepatocellular carcinoma and related cell lines. In addition, the high expression level of caveolin-1 was associated with venous infiltration and tumor metastasis, indicating that high caveolin-1 expression promoted the occurrence, progression, infiltration, and metastasis of tumors. Caveolin-1 can interact with transcription factor Fox M1 to promote epithelial-to-mesenchymal cell transformation and the metastasis of pancreatic cancer cells [19]. The downregulation of caveolin-1 expression can also inhibit STAT3 signaling pathways to block lung cancer cell metastasis [20]. High levels of caveolin-1 expression can also be seen in hand and neck squamous cell carcinoma and kidney cancer cells that have metastasized [21, 22]. Thus, caveolin-1 exhibits a high degree of upregulation in early tumor cells that metastasize to lymph nodes and is closely related to poor prognosis [23], indicating that in different stages of tumor development, the effects of caveolin-1 as an important molecule in the regulation of growth are also very different; that is, caveolin-1 is not only a tumor suppressor protein but also a transfer-related molecule.

In this study, the expression of caveolin-1 was significantly higher in prostate cancer than in benign prostatic hyperplasia. The positive rates of caveolin-1 expression in poorly, moderately, and well-differentiated prostate cancer were 80.0, 68.8 and 18.2%, respectively. The positive rates of caveolin-1 expression in T1-T2 and T3-T4 prostate cancer were 46.15 and 80.95%, respectively; there were no significant differences between the different clinical stages. In groups with different preoperative PSA levels (0–10 μg/L, 10–100 μg/L, and 100 μg/L), the positive rates of expression were 16.67, 68.18 and 68.42%, respectively; there were no significant differences among the three groups. It is assumed that the high expression of caveolin-1 in prostate cancer may be related to the interaction between the phosphorylation and dephosphorylation of scaffolding and multiple signaling pathways and to gene mutation and promoter methylation, but it is not related to serum PSA levels; these results suggest that caveolin-1 may play an important role in the progression of prostate cancer. The results from Mohammed, D.A. [24] also suggest that the expression of caveolin-1 in prostate cancer is significantly higher than in BPH and high-grade PIN, indicating that caveolin-1 plays an important role in the occurrence and metastasis of prostate cancer and is not related to the invasion of prostate cancer. Caveolin-1 expression is upregulated in the terminal development stages of various tumors and in various stages of urinary tumors, and it increases with tumor malignancy. The high expression of caveolin-1 is closely related to prostate cancer, promoting the occurrence, progression, and infiltration of the tumors. Possible mechanisms include gene site mutations, heterozygous deletions, and promoter hypermethylation. Paracrine caveolin-1 can promote the release of various growth factors by regulating their transcription, upregulating their mRNA and protein expression levels, stimulating tumor angiogenesis, and promoting tumor formation and proliferation. It can be used as a new biomarker for prostate cancer that can be used in the clinical diagnosis, treatment and prognosis of prostate cancer [25, 26].

How does caveolin-1 lose its inhibitory function as a tumor suppressor and instead promote tumor growth? It is generally believed that the mechanism of action may have three points: 1) point mutations [27]; 2) tyrosine phosphorylation [28]; and 3) serine phosphorylation. Under normal circumstances, caveolin-1 is an integrated membrane protein. However, if Ser is phosphorylated, caveolin-1 will be transformed into secreted proteins, which can improve its autocrine or paracrine function. Tahir et al. [29] demonstrated that androgen-insensitive prostate cancer cells could secrete caveolin-1 in autocrine or paracrine form and directly stimulate the growth and survival of prostate cancer cells. It is also believed that caveolin-1 activates the PI3-K-Akt signaling pathway in prostate cancer cells and promotes cancer cell metastasis by interacting with transfer molecules such as VEGF, TGF-β1 and FGF2 [30]. The absence of interstitial caveolin-1 expression is related to the activation of AKT, which leads to the upregulation of TGF-β1 and SNCG expression and results in the transfer of prostate cancer cells [31]. Caveolin-1 is upregulated in prostate cancer, and its high expression in most prostate cancer tissues can promote the proliferation of cancer cells and metastasis to lymph nodes. Caveolin-1 can be used as a biologically active molecule to promote tumorigenesis and tumor angiogenesis in the prostate cancer microenvironment. Caveolin-1 overexpression exists in primary and metastatic prostate cancer [32]. At present, caveolin-1 can be detected in the peripheral blood, and its expression level can be used to improve the diagnosis of prostate cancer and is related to prognosis.

## Conclusion

In this study, it was found that high caveolin-1 expression is closely related to prostate cancer after transurethral surgery and has a role in promoting tumorigenesis, development, and infiltration.

In conclusion, caveolin-1 is highly expressed in prostate cancer and is closely related to the pathological grade and clinical stage of prostate cancer. It may be a novel tumor marker for prostate cancer and has potential clinical value. The expression of caveolin-1 is not related to preoperative serum PSA levels.

## Abbreviations

BPH: Benign prostatic hyperplasia
Cav: Caveolin
FGF2: Factor fibroblast growth factor 2
MAPK: Mitogen-activated protein kinase
PCa: Prostate cancer.
PIN: Prostatic intraepithelial neoplasia.
PSA: Prostate specific antigen.
TNM: Tumor node metastasis.
VEGF: Vascular endothelial growth factor

## References

[1] Wong ECL, Kapoor A (2017). Epidemiology of prostate and kidney cancer in the aboriginal population of Canada: a systematic review. Can Urol Assoc J.

[2] Trama A, Botta L, Nicolai N, Rossi PG, Contiero P, Fusco M (2016). Prostate cancer changes in clinical presentation and treatments in two decades: an Italian population-based study. Eur J Cancer.

[3] Rainato G, Fabricio AS, Zancan M, Peloso L, Dittadi R, Barichello M (2016). Evaluating serum insulin-like growth factor 1 and insulin-like growth factor binding protein 3 as markers in prostate cancer diagnosis. Int J Biol Markers.

[4] Stephan C, Jung K, Miller K, Ralla B (2015). New biomarkers in serum and urine for detection of prostate cancer. Aktuelle Urol.

[5] Dickinson J, Shane A, Tonelli M, Connor GS, Joffres M, Singh H (2016). Trends in prostate cancer incidence and mortality in Canada during the era of prostate-specific antigen screening. Cmaj Open.

[6] Terada N, Akamatsu S, Kobayashi T, Inoue T, Ogawa O, Antonarakis ES (2017). Prognostic and predictive biomarkers in prostate cancer: latest evidence and clinical implications. Ther Adv Med Oncol.

[7] Sugie S, Mukai S, Yamasaki K, Kamibeppu T, Tsukino H, Kamoto T (2015). Significant Association of Caveolin-1 and Caveolin-2 with prostate Cancer progression. Cancer Genomics Proteomics.

[8] Yang HJ, Feng P, Wang L, Li ZC, Ma SP, Wang M (2015). Caveolin-1 mediates gene transfer and cytotoxicity of polyethyleneimine in mammalian cell lines. Mol Cell Biochem.

[9] Anwar SL, Wahyono A, Aryandono T, Haryono SJ (2015). Caveolin-1 in breast Cancer: single molecule regulation of multiple key signaling pathways. Asian Pac J Cancer Prev.

[10] Simpkins SA, Hanby AM, Holliday DL, Speirs V (2012). Clinical and functional significance of loss of caveolin-1 expression in breast cancer-associated fibroblasts. J Pathol.

[11] Garnett DJ (2016). Caveolae as a target to quench autoinduction of the metastatic phenotype in lung cancer. J Cancer Res Clin Oncol.

[12] Reis ST, Viana NI, Leite KR, Diogenes E, Antunes AA, Iscaife A (2016). Role of genetic polymorphisms in the development and prognosis of sporadic and familial prostate Cancer. PLoS One.

[13] Bocci G, Fioravanti A, Orlandi P, Di DT, Natale G, Fanelli G (2012). Metronomic ceramide analogs inhibit angiogenesis in pancreatic Cancer through up-regulation of Caveolin-1 and Thrombospondin-1 and Down-regulation of cyclin D1. Neoplasia.

[14] Shen X-J, Zhang H, Tang G-S, Wang X-D, Zheng R, Wang Y (2015). Caveolin-1 is a modulator of fibroblast activation and a potential biomarker for gastric Cancer. Int J Biol Sci.

[15] Ma W, Wang DD, Li L, Feng YK, Gu HM, Zhu GM (2014). Caveolin-1 plays a key role in the oleanolic acid-induced apoptosis of HL-60 cells. Oncol Rep.

[16] Friedrich T, Richter B, Gaiser T, Weiss C, Janssen KP, Einwächter H (2013). Deficiency of caveolin-1 in Apc(min/+) mice promotes colorectal tumorigenesis. Carcinogenesis.

[17] Xu J, Agyemang S, Qin Y, Aysola K, Giles M, Oprea G (2014). A novel pathway that links Caveolin-1 Down-regulation to BRCA1 dysfunction in serous epithelial ovarian Cancer cells. Enliven Challenges in cancer detection and therapy.

[18] Tse EY, Ko FC, Tung EK, Chan LK, Lee TK, Ngan ES (2012). Caveolin-1 overexpression is associated with hepatocellular carcinoma tumourigenesis and metastasis. J Pathol.

[19] Huang C, Qiu Z, Wang L, Peng Z, Jia Z, Logsdon CD (2012). A novel FoxM1-caveolin signaling pathway promotes pancreatic cancer invasion and metastasis. Cancer Res.

[20] Pancotti F, Roncuzzi L, Maggiolini M, Gaspericampani A (2012). Caveolin-1 silencing arrests the proliferation of metastatic lung cancer cells through the inhibition of STAT3 signaling. Cell Signal.

[21] Masuelli L, Budillon A, Marzocchella L, Mrozek MA, Vitolo D, Di Gennaro E (2012). Caveolin-1 overexpression is associated with simultaneous abnormal expression of the E-cadherin/α-β catenins complex and multiple ErbB receptors and with lymph nodes metastasis in head and neck squamous cell carcinomas. J Cell Physiol.

[22] Steffens S, Schrader AJ, Blasig H, Vetter G, Eggers H, Tränkenschuh W (2011). Caveolin 1 protein expression in renal cell carcinoma predicts survival. BMC Urol.

[23] Steiner I, Jung K, Miller K, Stephan C, Erbersdobler A (2012). Expression of endothelial factors in prostate cancer: a possible role of caveolin-1 for tumour progression. Oncol Rep.

[24] Mohammed DA, Helal DS (2017). Prognostic significance of epithelial/stromal caveolin-1 expression in prostatic hyperplasia, high grade prostatic intraepithelial hyperplasia and prostatic carcinoma and its correlation with microvessel density. J Egypt Natl Canc Inst.

[25] Bennett NC, Hooper JD, Johnson DW, Gobe GC (2014). Expression profiles and functional associations of endogenous androgen receptor and caveolin-1 in prostate cancer cell lines. Prostate.

[26] Nassar ZD, Moon H, Duong T, Neo L, Hill MM, Francois M (2013). PTRF/Cavin-1 decreases prostate cancer angiogenesis and lymphangiogenesis. Oncotarget.

[27] Lee H, Park DS, Razani B, Russell RG, Pestell RG, Lisanti MP (2002). Caveolin-1 mutations (P132L and null) and the pathogenesis of breast cancer: caveolin-1 (P132L) behaves in a dominant-negative manner and caveolin-1 (−/−) null mice show mammary epithelial cell hyperplasia. Am J Pathol.

[28] Lee H, Volonte’ D, Galbiati F, Iyengar P, Lublin DM, Bregman DB (2000). Constitutive and growth factor-regulated phosphorylation of caveolin-1 occurs at the same site (Tyr-14) in vivo: identification of a c-Src/Cav-1/Grb7 signaling cassette. Mol Endocrinol.

[29] Tahir SA, Yang G, Ebara S, Timme TL, Satoh T, Li L (2001). Secreted caveolin-1 stimulates cell survival/clonal growth and contributes to metastasis in androgen-insensitive prostate cancer. Cancer Res.

[30] Li L, Ren C, Yang G, Goltsov AA, Tabata K, Thompson TC (2009). Caveolin-1 promotes autoregulatory, Akt-mediated induction of cancer-promoting growth factors in prostate cancer cells. Mol Cancer Res.

[31] Wang S, Zhang C, Liu Y, Xu C, Chen Z (2014). Functional polymorphisms of caveolin-1 variants as potential biomarkers of esophageal squamous cell carcinoma. Biomarkers.

[32] Karam JA, Lotan Y, Roehrborn CG, Ashfaq R, Karakiewicz PI (2007). Caveolin-1 overexpression is associated with aggressive prostate cancer recurrence. Prostate.

